# Sleep Disturbances and Depression Are Co-morbid Conditions: Insights From Animal Models, Especially Non-human Primate Model

**DOI:** 10.3389/fpsyt.2021.827541

**Published:** 2022-01-25

**Authors:** Meng Li, Jieqiong Cui, Bonan Xu, Yuanyuan Wei, Chenyang Fu, Xiaoman Lv, Lei Xiong, Dongdong Qin

**Affiliations:** School of Basic Medical Sciences, Yunnan University of Chinese Medicine, Kunming, China

**Keywords:** depression, sleep, non-human primate, brain development, animal model

## Abstract

The incidence rates of depression are increasing year by year. As one of the main clinical manifestations of depression, sleep disorder is often the first complication. This complication may increase the severity of depression and lead to poor prognosis in patients. In the past decades, there have been many methods used to evaluate sleep disorders, such as polysomnography and electroencephalogram, actigraphy, and videography. A large number of rodents and non-human primate models have reproduced the symptoms of depression, which also show sleep disorders. The purpose of this review is to examine and discuss the relationship between sleep disorders and depression. To this end, we evaluated the prevalence, clinical features, phenotypic analysis, and pathophysiological brain mechanisms of depression-related sleep disturbances. We also emphasized the current situation, significance, and insights from animal models of depression, which would provide a better understanding for the pathophysiological mechanisms between sleep disturbance and depression.

## Introduction

Sleep is an essential physiological requirement for human and most animals. A mechanistic link is evident between sleep and depression at the molecular and neurophysiological level. The periodic regulation of awake and sleep requires the participation of many neurotransmitters, including excitatory neurotransmitters (such as acetylcholine) and inhibitory neurotransmitters (such as gamma aminobutyric acid, GABA). Abnormalities of these neurotransmitter systems not only lead to sleep-wake rhythm disorders, but also can contribute to developing depression. Depression and sleep disturbances are common co-morbid conditions (1, 2). More than 90% percent of patients with major depressive disorder will suffer from sleep disorders, which changed the patients' sleep structure. A further demonstration of the link between depression and sleep is that sleep can be improved by most clinically effective antidepressant drugs. Compared with lower mammals, the sleep of non-human primates (NHPs) is better comparable with that of humans. Recently, significant progress has been made in the study of using NHPs to establish depression models. Monitoring the sleep status of animals during modeling will help us further understand the role of sleep in the development of depression, and provide an objective biomarker for the early diagnosis, treatment, and efficacy evaluation.

## Sleep Structure and Related Neurotransmitters

Sleep is vital for human beings and most animals, and control mechanisms are embodied in all levels of biological organizations, from genes and intracellular mechanisms to cell population networks, and then to all central nervous systems, including systems that control movement, arousal, autonomic function, behavior, and cognition. Mammalian sleep is characterized by the periodic alternation of rapid eye movement sleep (REMs) and non-rapid eye movement sleep (NREMs). NREMs includes two stages: slow-wave sleep (SWS) and light sleep. In humans, SWS and REMs, which are the specific modes of potential electric field oscillations and neuromodulator activities, dominate the first half of the night and the latter half of the night, respectively (3).

The mutual transformation between sleep and wakefulness is caused by the excitation or inhibition of many neurotransmitters in the brain, which are released by sleep-promoting neurons in the anterior hypothalamus or sleep-inhibiting neurons in the lateral and posterior hypothalamus activity. These neurons release excitatory or inhibitory neurotransmitters to promote the brainstem to control the mutual transformation of wakefulness and sleep (2, 4).

The ascend arousal system mainly comes from a group of explicit cells with definite neurotransmitters. The arousal system actually consists of two channels (5). The ascending pathway to the thalamus is the first branch, which activates the thalamus and is essential to relay neurons for transmitting information to the cerebral cortex. The main sources of input from the upper brainstem to the thalamic-relay nucleus, the thalamic reticular nucleus, the pedunculopontine, and laterodorsally tegmental nucleus (PPT/LDT) are a couple of acetylcholine producing cell populations. The neurons in PPT/LDT discharge fastest during awake and REMs, and are often accompanied by cortical activation, loss of body muscle tone and active dreams. During NREMs, the activity of these cells is much lower. They are important for the input of reticular nucleus, because they are located between thalamic relay nucleus and cerebral cortex. It is very important for arousal that they can block the transmission between thalamic and cerebral cortex, thus acting as a gating mechanism. From the reticular structure and PPT/LDT, monoamine nervous system and parabrachial nucleus in the upper part of the brain stem, have more extensive input to the midline of thalamus and tabular nucleus. The laminar nucleus and midline nucleus are also considered to play a role in cortical arousal (5). Bypassing the thalamus and activating the neuronal pathway of the lateral hypothalamic area, basal forebrain (BF), and the whole cerebral cortex is the second branch of the ascending arousal system. This pathway, which covers noradrenergic locus coeruleus, serotonin dorsal nucleus, and median raphe nucleus, dopaminergic midbrain periaqueductal gray matter ventral and histaminergic nodule papillary neurons, is derived from monoamine neurons in the upper brainstem and caudal hypothalamus. Cortical input is increased by hypothalamic lateral peptidergic neurons (containing melanin concentrating hormone or orexin/retinol) and BF neurons (containing acetylcholine or GABA) (5). Lesions along this path, especially in the left hemisphere and the rostral midbrain, produce the most profound and lasting drowsiness and even coma. The neurons in each monoaminergic nucleus involved in this pathway discharge fastest during waking, slow down during NREMs and completely stop during REMs. It should be noted that all these ascending pathways pass through the regions at the junction of forebrain and brainstem. While, the descending pathways responsible for synchronizing phenomena still remain largely unknown at the brain-stem level.

The pathogenesis of sleep disorder is closely related to sleep-wake homeostasis, but the specific mechanism remains still unclear. During NREMs and REMs, different kinds of neurotransmitters are released in the brain. The interaction between aminergic neurons and cholinergic neurons at the meso-pontine junction leads each other to bring about the Ultradian rhythms alternation of REMs and NREMs. During NREMs, aminergic inhibition is decreased and cholinergic excitation is increased. At the onset of REMs, aminergic inhibition is turned off, cholinergic excitability reaches a peak, and other outputs are inhibited (2). When awake, the pontine aminergic system is tensely activated and the pontine cholinergic system is inhibited. In addition to aminergic and cholinergic neurons, other neurotransmitter systems are also involved in modulating REMs/NREMs alternation and may interact with aminergic and cholinergic systems (2, 6, 7). Extrinsically augmented dopaminergic neurotransmission can influence both REMs and NREMs cycles. Moreover, gamma-amino butyric acid and glutamate also affect the REMs/NREMs cycle (2).

In short, the growth and decline of these neurotransmitters promote the mutual transformation between sleep and wake. If these related neurotransmitters are released abnormally, it will cause sleep problems, such as difficulties in falling asleep and maintaining sleep state, changes of REMs latency, abnormal REMs behavior, and disturbed alternating pattern of REMs/NREMs.

## Clinical Characteristics and Related Neurotransmitters of Sleep Disorders in Depression

Depression is the main cause of the burden of mental health-related diseases in the world, and about 300 million people around the world are affected by depression (8). One aspect of efforts to understand depression focuses on its relationship with sleep. In many cases, the onset of depression is announced through sleep disorders, and sleep deterioration occurs before depression and manic episodes (9). There are many forms of sleep disorders reported in patients with depression. It may be only exhibited by the shortening of sleep time, but it also indicates a reduction in sleep efficiency. The latter is defined as the ratio of total sleep time to total time spent in bed over the night. Lack of sleep increases the risk of depressive episodes and depression relapses. Likewise, depression increases the risk of sleep disorders. However, the self-assessment of sleep quality in patients with depression is unreliable. Similarly, there are differences in the subjective and objective assessment of daytime alertness (10). This leads to bias in the evaluation of sleep efficiency.

Epidemiological investigations confirm that there is a closer relationship between insomnia and the onset of depression. It is reported that most patients often have insomnia and depressive episodes at the same time (11). Approximately 90% of major depressive disorder (MDD) patients have been found to suffer from sleep disorders, including initial insomnia, difficulty in sleep maintenance, non-restorative sleep, and early morning awakenings (12, 13). In reality, the most common subjective sleep complaints reported by depressed patients are insomnia (up to 88%) and hypersomnia (27%) (14). The insomnia and emotional symptoms are bidirectional correlated that poor sleep may precede the onset of depression, and depressive mood may also disrupt sleep patterns. In addition, patients with MDD are three times more likely to suffer from insomnia than those without (15, 16). Furthermore, fatigue, hypersomnia, and sleepiness are closely related to depressive symptoms (14). Many depressed patients complain about non-recovery sleep and excessive daytime sleepiness (16), and about 15% of patients report symptoms of daytime sedation and hypersomnia (17). However, these findings are inconsistent (16). Depression and hypersomnia are two conditions linked in a complex and bidirectional manner. In addition, many patients with depression call their complaints a combination of daytime sleepiness and nighttime anxiety.

Since the 1960s, polysomnography (PSG) sleep studies have repeatedly shown that depression is also associated with disrupted sleep architecture. These abnormalities include increase in RA (REMs activity) and RD (REMs density), as well as a decrease in REMs latency and SWS (18). During REMs, patients with depression often show short latency, prolonged cycle, and increased density (19). Disorders of REMs usually persist throughout the clinical episode, and it is considered to increase the possibility of recurrence, and may reduce the therapeutic effect (19–21). After antidepressant treatment, the number of REMs is decreased and the latency of REMs is increased. Most antidepressants inhibit REMs in patients and healthy volunteers (22).

The increase of serotonin content may be the main reason affecting REMs (23). Antidepressants that increased the contents of serotonin (5-HT) in synapses are effective inhibitors of REMs. 5HT1A agonists can be used as antidepressants and can significantly inhibit REMs (24). However, tryptophan depletion leads to a decrease in serotonin, which has been shown to reverse REMs inhibition caused by antidepressants (25). In addition, trazodone and nefazodone are also used as antidepressants because they have a strong antagonistic effect on serotonergic 5-HT2 receptors, which often promotes sleep and improves sleep continuity (26). The percentage of REMs was most significantly decreased in the early stage of treatment. Additionally, a subsequent study evaluated the changes in sleep structure of 20 patients with unipolar MDD after administration of sustained-release bupropion, and the results showed that 8 weeks of bupropion treatment significantly prolonged REMs latency, increased REMs activity and density in the first REMs period, which led to increased total REM density (27).

Glutamatergic and GABAergic neurons also play a role in the generation of REMs (28). Ketamine is a rapid-acting antidepressant (29), and AMPA-mediated increased neurotransmission is the basis of the antidepressant-like behavioral effects of ketamine (30, 31). The enhancement of AMPA receptor signal is participated in the pathophysiology and the mediation of ketamine-induced rapid antidepressant treatment (32, 33). Importantly, increased levels of ionic AMPA receptor could promote net synaptic strength and induce prolonged waking time in rodents and humans (34).

The REMs density of patients with depression continues to increase, which is regarded as an endophenotype. The reduction of the initial latency and the delta sleep ratio (DSR, the ratio of SWS between the first two NREMs episodes) of the rapid eye movement can be explained by cholinergic-aminergic imbalance (35). The monoaminergic inhibition of PPT/LDT cholinergic cells in patients with depression is weakened and/or the cholinergic-driven effect in pontine reticular formation is enhanced, resulting in an increase in REMs tendency and intensity.

The initiation and maintenance of NREMs also seem to be dependent on the role of monoamine neurotransmitters (26). Sedative antidepressants enhance SWS and prolong sleep duration. For instance, selective serotonin reuptake inhibitors (SSRIs) and non-sedating tricyclic antidepressants (TCA) can result in lighter sleep. In patients with depression, SWS and DSR tends to be low (36, 37). Compared with REMs latency, the measurement of SWS and DSR distribution may be a more reliable predictor of clinical response of antidepressant treatment and recurrence of depressive symptoms. Higher DSR may be more conducive to the treatment of depression (38). Some lines of evidence suggest that ketamine administration significantly increased the intensity of both SWS and DSR in humans and rats (39–41).

In addition, other types of antidepressants can also improve sleep. For example, antidepressants with anti-histaminergic action, such as mirtazapine and ipsapirone, act on their own receptors to support homeostatic maintenance of monoamine levels, block specifically monoamine receptors to enhance serotoninergic neurotransmission. Some patients' sleep can become better even after the first treatment of mirtazapine (42). However, increased levels of noradrenergic and dopaminergic neurotransmission, and raised activation of serotonergic 5-HT2 receptors can worsen the quality of sleep, which are also adverse effects of several antidepressants, such as serotonin and norepinephrine reuptake inhibitors, norepinephrine reuptake inhibitors, monoamine oxidase inhibitors (MAOI), SSRIs, and activated TCA (43). During REMs, monoaminergic neurons reduced significantly their discharge rate or stop their activity, but cholinergic neurons become highly active (44). However, MAOI increases the amounts of monoamine by preventing enzyme degradation and tends to cause the absence of REMs. One possible explanation is the antagonism of three receptors, namely H1 histamine or cholinergic receptor and postsynaptic 5HT2C receptor (26, 45). Therefore, most antidepressants alleviate depressed symptoms by improving sleep quality.

## Animal Models used in the Study of Sleep Disturbances and Depression

It is necessary to obtain the best animal model for studying disease in biomedical research. Validity of animal models depends on the extent to which how they can mimic human diseases. Researchers have made exogenous and endogenous animal models to simulate the symptoms of depressed patients and elucidate the mechanisms of antidepressant action, involving acute and chronic stress model, secondary depression model, and genetic model (46). Translation validity of animal models is the key to sleep disorders research. As shown in Table 1, zebrafish, mice, rats, cats, dogs, and monkeys are generally useful to develop animal models to study sleep disorders (49, 62, 98–101). Among them, the most used laboratory animals are mice and rats. However, they are quite different from humans as they are nocturnal and adopt a monophasic sleep schedule. While, humans follow a polyphasic sleep pattern and are very flexible in choosing the sleep time (80, 102). Similar to humans, more fragmented and polyphasic sleep patterns are observed in monkeys, and they are generally active during the day and sleep at night (84). In view of this, compared with other animals, the sleep pattern of monkeys is closer to that of humans.

**Table 1:** Comparison of different animal models used to study sleep disturbances and depression.

**Animal models**	**Main application fields**	**Pros**	**Cons**	**References**
Zebrafish	Molecular mechanisms of sleep/wake rhythm	Low cost; high gene-editing efficiency and relatively well-defined behavioral phenotypes	Not yet evaluated for depression-related sleep disturbances	(47–57)
Cat	Neuroendocrine mechanisms of sleep and sleep deprivation	Quantitative research of neurotransmitters	Not yet evaluated for depression and depression-related sleep disturbances	(58–60)
Dog	Sleep-wake cycle; narcolepsy; geriatric insomnia; obstructive sleep apnoea; sleep-associated epilepsy; and REMs disorder	Shared risks of many sleep disturbances with humans	More variable and fragmented sleep pattern; not yet evaluated for depression; and depression-related sleep disturbances	(61–67)
Rodents	Depression and sleep homeostasis; sleep structure; sleep-wake cycle; neurotransmitter receptor sensitivity and neuroendocrine stress response; as well as the effects of antidepressants on sleep	Low cost; easy to manipulate and gene-editing	Nocturnal animals; shorter durations of REMs and NREMs cycles	(68–82)
Non-human primates	Sleep-related neurobiology; neuroendocrine; and behavioral pharmacological studies	Highly similar to humans in brain structure, behavior, metabolism, sleep characteristics, and circadian rhythms	Difficult to directly measure mood or thoughts; limited behavioral screening tools; and lack of the effects of antidepressants on sleep	(83–97)

## Rodent Models

Rodents are more usual choice of preclinical models to develop new pharmacological and non-pharmacological strategies. In the study of sleep deprivation, rodents (i.e., rats, mice) and humans have many similarities in sleep electroencephalogram (EEG) and sleep structure (103). External stressors or risk factors of diseases can affect the number or pattern of REMs (22, 104, 105). In humans, REMs latency is negatively correlated with the severity of depression (37). In rodents, changes in the REMs can precede those of other sleep/wake stages. For example, mice that were applied to water immersion for 2 h and restraint stress exhibited an immediate reduction in REMs (106).

As for the effect of stress on rodents' total sleep time, the primary stressors are immobilization and mild electrical shock. Immobilization increased the time spent in SWS and REMs, while electrical shock resulted in a decrease in total sleep time and total REMs time (107). Similarly, fear conditioning paradigms can also induce a decrease in REMs during both the shock training and cue exposure (104). Chronic unpredictable mild stress can lead to changes in the amplitude of both physiological (i.e., locomotion, temperature) and molecular circadian rhythm, which may cause depressive-like behaviors (108).

Continuous light exposure (LL) increases depressive-like behavior in mice, and light exposure at night (LAN) can lead to depressive-like behavior in diurnal rodents, such as grass rats and hamsters (109–111). This may be because LL brings about the interrupted rhythm of locomotion, temperature, and hormonal release, causing the disruption of circadian rhythm, and increases of NREMs during the rest period and REMs in the active period (112, 113).

For social species such as rats and mice, repeated fighting and/or defeat may be a more natural source of stress. Often, the consequences of chronic social defeat stress (CSDS) can persist until the termination of the stressors, which makes it a particularly attractive method to model stress-related psychiatric illnesses (114). Previous studies have found that CSDS has a direct effect on subsequent sleeping. Specifically, it can increase both the total time of REMs and NREMs, as well as the density of NREMs. However, the number of REMs is significantly decreased in the first few hours after conflict (114, 115). Another experiment also reported a brief increase in REMs time following 10 days of social conflict, but no changes in SWS were detected (115, 116). Interestingly, there was no difference in NREMs and slow-wave activity between winner and loser, suggesting it is a consequence induced by the conflict process.

## Non-Human Primate Models

NHPs bridge the gap between rodents and humans (117). Like humans, NHPs have stable sleep at night and some nap during the day. Many kinds of non-human primates, such as baboon, Kenya baboon, South African ape, macaque, cynomolgus monkey, Pada monkey, lemur, and chimpanzee, can be used to study sleep. By comparing the sleep of non-human primates, researchers generally believe that chimpanzee, olive baboon, and rhesus monkey are better model animals. In monkeys, four sleep EEG patterns can be easily identified. Due to its stable and perfect sleep architecture, macaque has become the best model to study the biological characteristics of human sleep (118–121). During the whole night, macaques experienced the alternation of awake, NREMs and REMs, and the total sleep time of rhesus monkey is about 10.5 h per day. It has been found that REMs time accounts for 23%, each of which lasts about 6 min and occurs every 51 min. In the early stage, it is mainly deep sleep, such as SWS. While, it is mainly REMs in the late stage of sleep. These sleep characteristics are very similar to humans that the interval of this cycle is about 90 min. Nevertheless, in rats, the interval is only 13 min. Like humans, obvious theta waves cannot be recorded in the hippocampus during macaques' sleep (122, 123).

EEG is a common method in sleep research, which can provide objective functional indexes for sleep (124). Although EEG can be performed in constrained animals under laboratory conditions, this technique is invasive. Even if it is minimally invasive, it also needs to drill holes in the skull and implant electrodes directly on the brain. PSG plays a cornerstone role in long-term recording of sleep, and has become the gold standard to evaluate sleep disorders. The recorded parameters include the brain activity (EEG), electrooculogram (EOG), expanded EEG montages, and transcutaneous or end-tidal capnography waveform, which are used to comprehensively monitor the normal and abnormal physiological indicators during sleep (125). However, an important limitation of PSG is that it requires electrodes and sensors (126). In addition, expensive and long-term recording intervals may be another limitation. Obviously, these are difficult and impossible to use in freely moving monkeys. A recent study compared videography and actigraphy methods in 10 cynomolgus monkeys during seven nights. It is verified that in the sleep study of NHPs, actigraphy can be regarded as a supplementary technique for routine EEG and/or video analysis to measure the sleep (127).

Researchers have used NHPs to make great efforts in the research of depression. It has been demonstrated for the first time that long-term intracerebroventricular administration of IFN-α (5 days/week for 6 weeks) can induce the monkeys showing considerable depressive-like symptoms with changes in the concentration of monoamine metabolites (128). The relationship between early adversity, chronic stress and depression was also investigated in adolescent monkeys. Eight male rhesus monkeys went through unpredictable chronic stress for 2 months and exhibited significant depression-like behaviors (88). The mechanisms underlying stress-induced depression were also explored in monkeys, and it was found that cortisol hypersecretion interacted with stress to accelerate the development of depressive behaviors (129).

In addition, researchers have employed NHPs animal model to make many beneficial explorations on the association between light deprivation and depression. The results showed that monkeys could develop the main symptoms of seasonal affective disorder under short lighting conditions (130). Analogous to depression in humans, sleep disorders have been also reported in spontaneous depressed monkeys (86). Notably, only the hypersomnia subgroup of spontaneously depressed monkeys shows a specific response to acute ketamine administration, characterized as extended wakefulness and shortening of nocturnal sleep. As a matter of fact, these changes are similar to sleep deprivation in depressed patients, suggesting alternation of nocturnal sleep pattern might help improve depressed mood (86, 119, 131).

## Conclusion and Perspectives

There is increasing evidence that sleep plays a causal role in emotional processing and regulation (132). Depression and sleep disturbances are common co-morbid conditions, and almost all depressed patients show some types of sleep disturbances (133, 134). Most antidepressants can change sleep, and the effects appear to be most significant and consistent on REMs (135). Selective REMs deprivation (such as forced awakenings) can produce an antidepressant effect, illustrating the closer association between REMs regulation and mechanisms involved in the development of depression (136). Some neurotransmitter reuptake inhibitors can alleviate depression by suppressing REMs through inhibition of serotonin and norepinephrine reuptake (26). However, many questions remain to be answered in future studies. Firstly, in previous studies, it was found that the effects of antidepressants on sleep initiation and maintenance were inconsistent. Secondly, the mechanism of different effects of antidepressants on sleep continuity is unclear. In rodent experiments, many paradigms of chronic stress have been used to simulate the pathogenesis of human depression, but it is hard to provide a unified description about the impact of chronic stress on sleep patterns. In fact, in addition to the types of stress, the number and persistent time are also important factors for stress responses, which must be carefully considered. NHPs are suitable animal models for experiments related to sleep, however, the study of depression and sleep disorders is far from enough. Although researchers have made continuous efforts and good progress in relevant animal models, it must be recognized that there are deficiencies.

In any way whatever, the research on animal models of sleep disorders provides a good clue and basis for clinical diagnosis and treatment of depression. NHPs are considered as a further valuable and translational animal model, which is necessary for sleep and related diseases (137, 138). It is also an important entry point for increased efforts dedicated to collaborative translational endeavors.

## Author Contributions

DQ, LX, XL, and ML designed the structure. ML, JC, BX, YW, CF, and DQ wrote the first draft of the manuscript. DQ, LX, and XL supervised and revised the final version of the manuscript. All authors contributed substantially to the scientific process, writing of the manuscript, and have approved the final version of the manuscript being submitted.

## Funding

This work was supported by the National Natural Science Foundation of China (31960178, 82074421, and 82160923), the Applied Basic Research Programs of Science and Technology Commission Foundation of Yunnan Province (2019FA007), the Joint Project of Applied Basic Research of Yunnan University of Chinese Medicine & Yunnan Provincial Science and Technology Department [2019FF002(-001)], Yunnan Provincial Department of Education Science Research Fund Project (2020Y0203), the Key Realm R&D Program of Guangdong Province (2019B030335001), the China Postdoctoral Science Foundation (2018M631105), and the Yunnan Provincial Academician and Expert Workstation (202005AF150017 and 2019IC051).

## Conflict of Interest

The authors declare that the research was conducted in the absence of any commercial or financial relationships that could be construed as a potential conflict of interest.

## Publisher's Note

All claims expressed in this article are solely those of the authors and do not necessarily represent those of their affiliated organizations, or those of the publisher, the editors and the reviewers. Any product that may be evaluated in this article, or claim that may be made by its manufacturer, is not guaranteed or endorsed by the publisher.
